# Pleiotropic Effect of AccD5 and AccE5 Depletion in Acyl-Coenzyme A Carboxylase Activity and in Lipid Biosynthesis in Mycobacteria

**DOI:** 10.1371/journal.pone.0099853

**Published:** 2014-06-20

**Authors:** Bernardo Bazet Lyonnet, Lautaro Diacovich, Matías Cabruja, Fabienne Bardou, Annaïk Quémard, Gabriela Gago, Hugo Gramajo

**Affiliations:** 1 Laboratory of Physiology and Genetics of Actinomycetes, Instituto de Biología Molecular y Celular de Rosario (IBR-CONICET), Facultad de Ciencias Bioquímicas y Farmacéuticas, Universidad Nacional de Rosario, Rosario, Argentina; 2 CNRS, IPBS (Institut de Pharmacologie et de Biologie Structurale), Département Tuberculose et Biologie des Infections, Toulouse, France; 3 Université de Toulouse, UPS, IPBS, Toulouse, France; University of Padova, Medical School, Italy

## Abstract

Mycobacteria contain a large variety of fatty acids which are used for the biosynthesis of several complex cell wall lipids that have been implicated in the ability of the organism to resist host defenses. The building blocks for the biosynthesis of all these lipids are provided by a fairly complex set of acyl-CoA carboxylases (ACCases) whose subunit composition and roles within these organisms have not yet been clearly established. Previous biochemical and structural studies provided strong evidences that ACCase 5 from *Mycobacterium tuberculosis* is formed by the AccA3, AccD5 and AccE5 subunits and that this enzyme complex carboxylates acetyl-CoA and propionyl-CoA with a clear substrate preference for the latest. In this work we used a genetic approach to unambiguously demonstrate that the products of both *accD5* and *accE5* genes are essential for the viability of *Mycobacterium smegmatis*. By obtaining a conditional mutant on the *accD5-accE5* operon, we also demonstrated that the main physiological role of this enzyme complex was to provide the substrates for fatty acid and mycolic acid biosynthesis. Furthermore, enzymatic and biochemical analysis of the conditional mutant provided strong evidences supporting the notion that AccD5 and/or AccE5 have an additional role in the carboxylation of long chain acyl-CoA prior to mycolic acid condensation. These studies represent a significant step towards a better understanding of the roles of ACCases in mycobacteria and confirm ACCase 5 as an interesting target for the development of new antimycobacterial drugs.

## Introduction

*Mycobacterium tuberculosis*, the causative agent of tuberculosis, remains one of the world's top infectious killers, with an estimated 8.7 million new tuberculosis cases and 1.4 million fatalities each year [1]. The ongoing AIDS pandemic has developed a deadly synergy with tuberculosis and the emergence of multi-drug-resistant (MDR) and extensively drug resistant (XDR) *M. tuberculosis* contributed further to deteriorate the control of tuberculosis in developing countries [2], [3]. In this context, the effective control of this major public health problem requires the identification of novel drug targets suitable for the development of new anti-mycobacterial drugs.

Mycolic acids, α-alkyl, β-hydroxylated fatty acids of unusual chain length (C_60_–C_90_), are essential and major constituents of the lipid rich envelope of *M. tuberculosis* and are located in the outer membrane (or mycomembrane). They play important roles in the reduced cell wall permeability [4], [5], virulence and persistence [6]–[9], and acid-fastness of *M. tuberculosis* [10]. Extensive enzymology and genetic evidences concerning the biosynthesis of mycolic acids and the other mycobacterial complex lipids are available; although several steps remain obscure [11]. In particular, the information regarding the metabolic pathways involved in the biosynthesis of the elongation units (malonyl-CoA, methylmalonyl-CoA and long-chain carboxyacyl-CoA) used by Fatty Acid Synthases (FASs) type I and II and PKSs *in vivo* is still very limited.

In mycobacteria, the α-carboxyacyl-CoAs utilized as elongation units in the biosynthesis of membranes lipids, are the product of different acyl-CoA carboxylase (ACCase) complexes. These enzymes, whose molecular composition appear to be unique within the phylum *Actinobacteria* [12]–[14], are attractive targets for the development of new and specific antimycobacterial agents. The reaction catalyzed by the ACCases occurs in two catalytic steps [15]; in the first step, the biotin carboxylase component (BC) couples carbonate to a biotin residue attached to a biotin carboxyl carrier protein (BCCP) to form carboxybiotin. In the second step, the carboxyltransferase component (CT) transfers the carboxyl group from biotin to the acyl-CoA and generates the corresponding α-carboxyacyl-CoA. In actinomycetes, the ACCases consist of two large polypeptides: an α-subunit that contains the BC and the BCCP domains and a β-subunit that contains the CT domain [14]. X-ray crystallography, mutagenesis, and enzyme kinetics studies demonstrated that the β-subunit is the major determinant of substrate specificity of these enzymatic complexes [12], [16], while the α-subunit can be shared by ACCase complexes with different substrate preferences. In some cases, a third subunit, called å, is essential for holo complex full activity; this å subunit is so far a unique feature of actinomycetes ACCases [12], [17].

*M. tuberculosis* has several genes coding for different ACCase subunits in its genome: three α subunits (*accA1–3*), six β subunits (*accD1–6*) and one å subunit (*accE5*) [18]. To date, only two ACCase complexes from *M. tuberculosis* have been thoroughly characterized at the biochemical level *in vitro*. ACCase 5 was reconstituted from the biotinylated α subunit AccA3, the CT β subunit AccD5 and the å subunit AccE5 [17], [19], and ACCase6 was reconstituted from the AccA3 and AccD6 subunits [20]. Kinetics studies indicated that both holo complexes accept acetyl- and propionyl-CoA as substrates. However, ACCase 5 has preference for propionyl-CoA, suggesting that its main physiological role could be to provide methylmalonyl-CoA for the biosynthesis of multimethyl-branched fatty acids [17]. In contrast, ACCase 6 carboxylates acetyl- and propionyl-CoA with similar efficiencies, and genetic studies conducted on an *accD6* conditional mutant in *M. smegmatis* suggested that its physiological role is to provide malonyl-CoA for fatty acid and mycolic acid biosynthesis [21], [22]. A third ACCase complex, the so-called “long chain acyl-CoA carboxylase”, has been less characterized at the biochemical and structural levels; however there are some genetic and biochemical evidences suggesting that this enzyme complex could have a fairly complex subunit composition in actinomycetes. The first genetic studies that generated information regarding the long chain ACCase subunit composition were carried out in *Corynebacterium glutamicum*. The generation of an *accD4* mutant in this organism resulted in a lack of mycolic acid production and in the absence of tetradecylmalonic acid, suggesting that AccD4 is the β component (CT) of the long-chain ACCase that generates the C_16_ α-carboxy acyl-CoA that, after its condensation with the meromycolyl-AMP forms the corynomycolic acid α-branch [23]. An additional support to this conclusion came from the genomic organization of *accD4* which is found clustered with and transcribed in the same orientation as *pks13* and *fadD32*, the genes encoding for the enzyme system involved in the final step of biosynthesis of mycolic acids [24]. Interestingly, the same genetic organization occurs for the orthologues of *accD4*, *pks13* and *fadD32* in mycobacteria suggesting that AccD4 of this organism plays the same role that its counterpart in *C. glutamicum* [23], [24]. Phylogenetic analyses showed that AccD4 is present exclusively in mycolic acid containing bacteria, confirming a specific role for this CT subunit in the biosynthesis of these complex lipids [17]. Furthermore, co-immunoprecipitation and copurification studies, carried out in cell-free extracts of *M. smegmatis*, demonstrated that AccD4 interacts with both AccA3 and AccD5 subunits [23], suggesting that the ACCase4 complex is formed by the α subunit AccA3 and two β subunits, AccD4 and AccD5. However, so far, there are no concluding biochemical evidences regarding the exact subunit composition of an active long chain acyl-CoA carboxylase complex. Furthermore, the ACCase5 complex has only been studied *in vitro* and the question of its physiological role in mycobacteria still needs to be addressed.

In this work, we present a genetic and physiological characterization of the ACCase 5 complex of mycobacteria, based on the analysis of an *accD5-accE5* conditional mutant generated in *M. smegmatis*. The results demonstrate that *accD5* and *accE5* are both essential for the viability of this bacterium and that the main physiological function of the ACCase5 complex in mycobacteria is to generate malonyl- and methylmalonyl-CoA for lipid biosynthesis. Consistently, the AccD5-E5 depletion in a conditional mutant has a strong impact on the pool of malonyl-CoA and also on the *de novo* fatty acid and mycolic acid biosynthesis.

## Materials and Methods

### Bacterial strains, culture, and transformation conditions

*Escherichia coli* strain DH5α [25] was used for routine subcloning and was transformed according to [26]. The transformants were selected on LB media supplemented with the appropriate antibiotics: 50 µg kanamycin (Km) ml^−1^, 20 µg gentamicin (Gm) ml^−1^, 20 µg streptomycin (Str) ml^−1^ and/or 50 µg apramycin ml^−1^. *M. smegmatis* mc^2^155 is an electroporation proficient mutant of mc26 [27]. Liquid cultures *of M. smegmatis* were grown at 37°C (or 42°C for D5SCO6) in Middlebrook 7H9 media supplemented with 0.2% glycerol, 0.03% tyloxapol and the appropriate antibiotics at the following concentrations: Km 15 µg ml^−1^, Gm 5 µg ml^−1^, Apra 50 µg ml^−1^ and Str 10 µg ml^−1^. The same antibiotic concentrations were used in solid LB media. ATc was dissolved in DMSO and added to the media at a final concentration of 200 ng ml^−1^.

### DNA manipulations and plasmid construction

Isolation of plasmid DNA, restriction enzyme digestion and agarose gel electrophoresis were carried out by conventional methods [26]. Genomic DNA of *M. smegmatis* was obtained as described by [28].

### Construction of *M. smegmatis* D5SCO6 strain

For the first recombination step, the temperature sensitive plasmid pPR-FD5, harboring a disrupted copy of the *accD5* gene (*accD5::aphA-3*), was introduced into *M. smegmatis* by electroporation and one of the Km^r^ transformants was plated at 42°C to promote plasmid recombination. Southern Blot analysis of the chromosomal DNA from nine Km^r^ XylE^+^ colonies indicated that two of them resulted from a single recombination event in the chromosomal copy of *accD5*; the other seven clones most probably arose from illegitimate recombination. One of the *M. smegmatis* colonies containing the correct integration of pPR-FD5 was selected for further analysis and named D5SCO6 (Fig. S2).

### Construction of *M. smegmatis* D5 MUT conditional mutant

To construct a conditional *accD5* mutant, we generated an *accD5* merodiploid strain by introducing pBB25 into D5SCO6. Plasmid pBB25 expresses *accD5* from the P*_tr_* promoter [29]. One of the Km^r^ Apra^r^ transformants obtained, called D5SCO6/pBB25, was grown at 37°C in LB-Km-Apra and plated on LB-Km-Apra-Suc plates at 37°C. At least 30% of the colonies grown in these conditions were also white after spraying with catechol, indicating that they had undergone intrachromosomal allelic exchange. One of the colonies obtained, named D5DCO/pBB25, was further transformed with plasmids pFra42B (Str^r^) yielding the strain D5 MUT. In this strain, the expression of *accD5-accE5* can be controlled by the addition of ATc. The chromosomal organization of the conditional mutant obtained was confirmed by southern blot. The isogenic strain ISO-D5, used as control, was obtained transforming *M. smegmatis* mc^2^155 with the plasmids pBB25 and pFra42B.

### Southern blot analysis

10 µg of genomic DNA was digested overnight with an excess of *Eco*RI, and the fragments were separated by electrophoresis in a 0.7% agarose gel. Southern blotting was carried out in 20× SSC using Hybond-N+ membrane (Amersham). The probe consisted of a 390 bp fragment amplified with oligonucleotides FD5-Fw-Xba (5′-TCTAGACCAGTGGCAGCAAAAGA-3′) and N-D5-Ms-UP (5′- GCCAGCTTGCCCGCAGTGGTGTGGATG-3′). The Prime-a-Gene labeling system (Promega) and 5 µCi (185 MBq) of [α-^32^P]dATP were used to label the probe. Pre-hybridization and hybridization were carried out at 65°C using 5× SSC, 5× Denhardt's solution and 0.5% SDS. Serial 15 min washes were performed at 65°C as follows: two washes with 2×SSC/0.1% SDS and two washes with 1× SSC/1% SDS. The filter was developed and digitalized with a Storm 840 scanner (Amersham).

### ACCase enzyme assays

ACCase activities in cell-free extracts were measured by following the incorporation of radioactive HCO_3_^−^ into acid non-volatile material, as previously described [12]. Substrate concentrations were 0.5 mM for acetyl-CoA and propionyl-CoA and 100 µM for C_16_-CoA. One unit of enzyme activity catalyzed the incorporation of 1 mmol ^14^C into acid-stable products min^−1^.

### Assay of NADP-dependent malic enzyme

NADP-dependent malic enzyme activity was determined spectrophotometrically at 30°C using a standard reaction mixture containing 50 mM Tris-HCl, pH 8.0, 10 mM MgCl_2_, 0.5 mM NADP^+^ and 30 mM L-malate in a final volume of 0.5 ml. The reaction was started by the addition of cell-free extracts, and the increase in A_340_ was recorded. One unit (U) is defined as the amount of enzyme that catalyses the formation of 1 µmol NADPH min^−1^ under the specified conditions.

### RNA techniques

#### RNA extraction

RNA was extracted from mid-log-phase cultures of *M. smegmatis* using the SV total RNA isolation system (Promega) and treated when needed with RQ1 RNase-Free DNase (Promega).

#### RT-PCR

To assess the operon nature of *accD5* and *accE5*, RNA was extracted from mid-log-phase cultures of *M. smegmatis* mc^2^155. Reverse transcription reaction was carried out using SuperScript III Reverse Transcriptase (Invitrogen) and random primers. PCR amplification of the intergenic regions on cDNA were performed using specific primers on *accD5* and *accE5*: D5-MS-Final (5′-CGTCGCACATATGCGGCTACGTG-3′) and FD5-Rv-Hind (5′-AAGCTTGACGACGCCGAACC-3′)

### Protein methods

Proteins were analysed by SDS-PAGE [30]. Protein contents were determined using Quant-iT Protein Assay Kits and Qubit fluorometer (Invitrogen). For western blot analysis, proteins were electroblotted onto nitrocellulose membranes (Bio-Rad) and probed with 1∶1000 dilution of polyclonal anti-AccD5 and anti-KasA. Immune complexes were visualized using horseradish peroxidase conjugated secondary antibodies against rabbit-IgG (for AccD5 detection) or rat-IgG (for KasA detection). Antiserum against AccD5 was elicited in rabbits. Antiserum against KasA was a kind gift of Dr. L. Kremer.

### Acyl-CoA content analysis

Acyl CoAs were extracted using a modification of the method described in Sun *et al* 2006 [31], from aliquots containing the same number of cells. The resulting acyl CoAs were separated on a Agilent 1200 SL (Agilent Corporation, Santa Clara, CA, United States) with a Thermo Scientific Hypersil GOLD C18 column with dimensions 2.1 mm×150 mm×3 um (Thermo Fisher Scientific, Waltham, MA, United States). High resolution mass spectrometry was performed with a Bruker micrOTOF-QII a Q-TOF instrument (Bruker Corporation, Billerica, MA, United States) with an electrospray ionization source (ESI; Bruker Corporation, Billerica, MA, United States). Data analysis was performed using the Bruker Daltonics's Compass DataAnalysis (Bruker Corporation, Billerica, MA, United States) software. Data were imported into datasheets for quantitative and statistical analysis.

### Analysis of fatty acids and mycolic acids

*De novo* fatty acid and mycolic acid biosyntheses were followed by labeling 5 ml culture aliquots with 1 µCi/ml [1-^14^C]-acetate (specific activity: 55.3 mCi/mmol; Perkin Elmer) for 1 h at 37°C. Fatty acid and mycolic acid methyl esters were extracted from samples containing equivalent amounts of bacteria as described by [32]. The resulting solution of FAMEs and MAMEs was assayed for radioactivity in a Beckman liquid scintillation counter and then subjected to TLC using silica gel plates (5735 silica gel 60F254; Merck). Samples were normalized by culture OD or total cpm and developed in hexane∶ethyl acetate (9∶1, v/v). Autoradiograms were produced by overnight exposure to Kodak X-Omat AR film to reveal ^14^C-labelled FAMEs and MAMEs.

### Statistical analysis

Data are reported as arithmetic means of the results obtained from three independent experiments ± standard deviations. Statistical significance was calculated using ANOVA and Mann Whitney test. Statistical significance was accepted at *P*<0.05.

## Results

### The *accD5-accE5* operon is essential for the viability of *M. smegmatis*

Bioinformatic, biochemical and structural analyses indicated that ACCase 5 complex of *M. tuberculosis* has a propionyl-CoA/acetyl-CoA carboxylase (PCC/ACC) activity *in vitro* [17], [33]. This complex is formed by the biotinylated α subunit AccA3, the carboxyltransferase β subunit AccD5 and the small å subunit AccE5. Studies carried out by high-density mutagenesis in *M. tuberculosis* predicted that the three genes were essential for the viability of this microorganism [34], [35].

**Figure 1:**
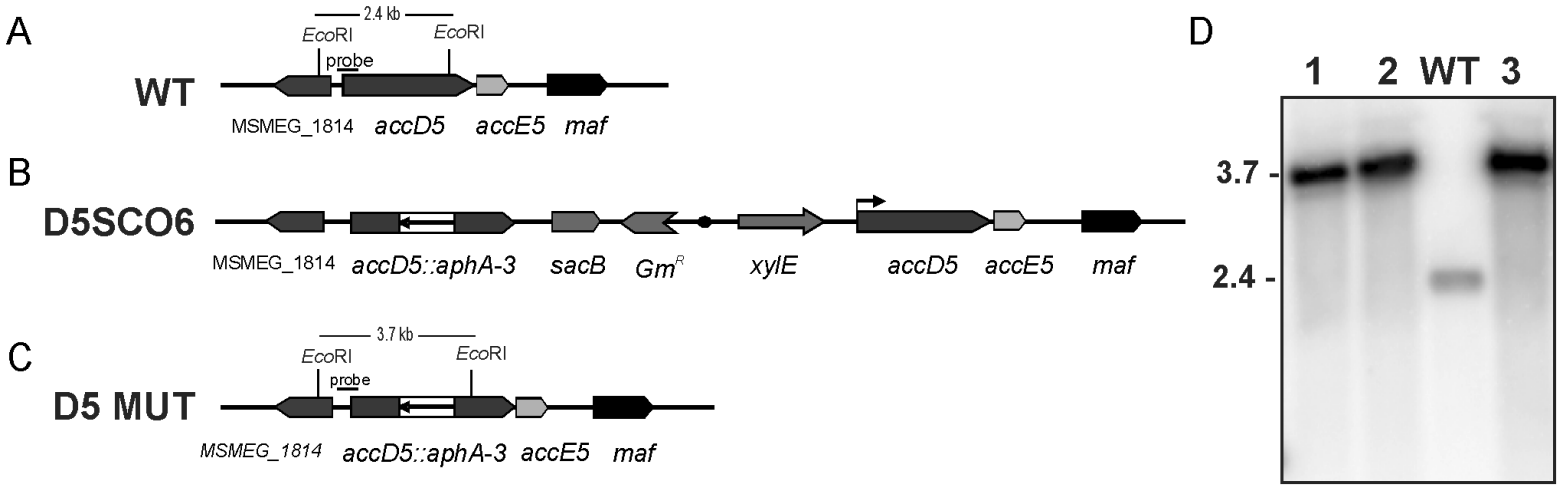
Allelic exchange of the *accD5* locus of *M. smegmatis*. A-C. Genetic organization and partial restriction maps of the *accD5* chromosomal region of (A) the wild-type strain mc^2^155, (B) the single crossover strain D5SCO6, and (C) the *M. smegmatis* D5DCO conditional mutant. D) Southern blot analysis of *M. smegmatis accD5* mutants. Three conditional mutants were picked at random (lanes 1–3), and their chromosomal DNA digested with *Eco*RI and probed for hybridization with a 390 bp labeled fragment corresponding to the 5′ region of *accD5*. *M. smegmatis* mc^2^155 DNA was included as a control (WT). Molecular masses are indicated in kilobases.

To gain insight about the metabolic relevance of ACCase 5 complex in mycobacteria, we decided to construct KO mutants in the genes encoding for two of its subunits, AccD5 and AccE5, in *M. smegmatis*. Before carrying out the specific mutations, we studied by RT-PCR if *accD5* and *accE5* were part of a single transcriptional unit. As shown in Figure S1, by using primers which hybridize with the 3′ ends of *accD5* and *accE5*, a PCR fragment that included the intergenic region of these two genes was amplified, confirming that they were part of a single operon. Therefore, we decided to disrupt *accD5* with an antibiotic resistant cassette, which would have a polar effect on the expression of *accE5*, and result in a double KO mutant. Thus, we replaced the wild-type *accD5* gene by the *accD5::aphA-3* mutant allele, using a classic two-step homologous recombination approach [36]. The single-crossover (SCO) strain D5SCO6 was obtained but attempts to obtain the second recombination event, leading to the exchange of the wild type chromosomal *accD5* by the mutant allele were unsuccessful, strongly suggesting that *accD5* and/or *accE5* genes were essential for *M. smegmatis* survival. To further dissect the essentiality of these genes, the same screening was performed in a D5SCO6 strain containing the plasmid pCGFD5 which harbors the *accD5-accE5* genes under the transcriptional control of the native promoter (Fig. 1A–B) and two independent isolates of D5SCO6/pCGFD5 were subjected to a second crossover analysis in LB-Km-Suc plates. In both cases more than 50% of the colonies that grew under these conditions were also white after spraying with catechol, indicating that they had undergone intra-chromosomal allelic exchange. Three individual colonies of the putative mutants were studied by Southern blot analysis, which confirmed that they all had the expected allelic exchange at the chromosomal *accD5* locus (Fig. 1C–D); one of these mutant strains was selected and named D5DCO2.

**Figure 2:**
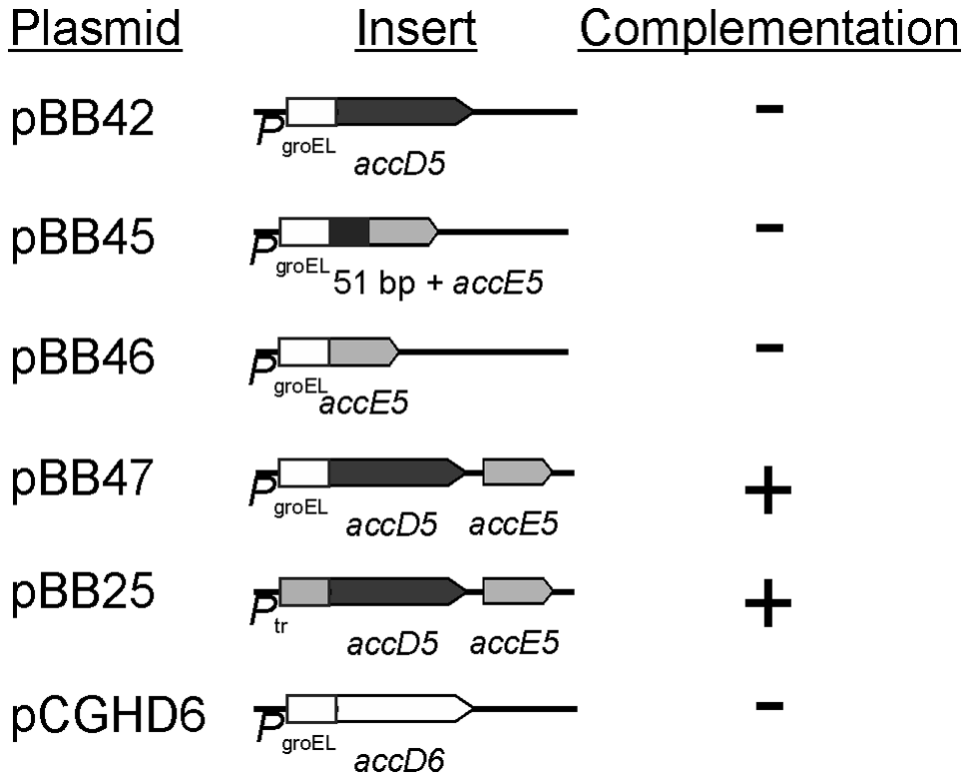
Complementation of the *accD5-accE5* conditional mutant. Schematic representation of the pMP395 derived constructions used in the complementation studies.

To determine which gene(s) of the *accD5-accE5* operon was (were) essential for the viability of *M. smegmatis*, five plasmids were constructed based on the expression vector pMP395 [37] (Fig. 2), and introduced into the D5SCO6 strain. These plasmids contained either *accD5*, *accE5* or *accD5-accE5* from *M. smegmatis*, under the control of P*_groEL_* promoter (Fig. 2). We also tested a construct coding for a longer version of *accE5* from *M. smegmatis* as proposed by Oh *et al* [19]. Isolates of D5SCO6 with the corresponding plasmids were screened for the occurrence of the second crossover event in LB-Km-Suc plates. None of the thousands Km^r^ Sac^r^ colonies screened exhibited the white phenotype after catechol spraying when the transformed plasmid contained either *accD5* or *accE5* (pBB42, pBB45, or pBB46) (Fig. 2), strongly suggesting that both genes are independently essential for *M. smegmatis* growth. Furthermore, when the expression vector contained both *accD5* and *accE5* genes from *M. smegmatis* (pBB47), more than 70% of the Km^r^ Sac^r^ colonies were white after spraying with catechol (Fig. 2), indicating that they had undergone intra-chromosomal allelic exchange and demonstrating than both genes are essential for the viability of this organism. The same results were obtained when the D5SCO6 strain was complemented with plasmid pBB25, which contains the *accD5-accE5* genes under the control of the P*_tr_* promoter [38]. Finally, to study whether constitutive and strong expression of the β subunit AccD6 could compensate the loss of the acetyl-CoA carboxylase activity of ACCase 5 and replace AccD5, we tried to complement the *accD5* mutation with a plasmid expressing *accD6* from *M. smegmatis* (pCGHD6), but we could not obtain the second recombination event, suggesting that ACCase 5 and 6 have distinct roles in mycobacteria.

### Construction and characterization of an *accD5*-*accE5* conditional mutant

Although the genetic studies performed above showed the essentiality of the β and å subunits of the ACCase 5 complex for the survival of *M. smegmatis*, the recombinant strain D5DCO2 did not practically allow investigations on the *in vivo* role of the enzyme complex. To address this question, we set up the construction of a conditional *accD5-accE5* mutant in *M. smegmatis* mc^2^155. For this, we used an adaptation of the approach developed by Boldrin *et al* [38], based on two different repressors (TetR and Pip) encoded at the chromosomal level.

**Figure 3:**
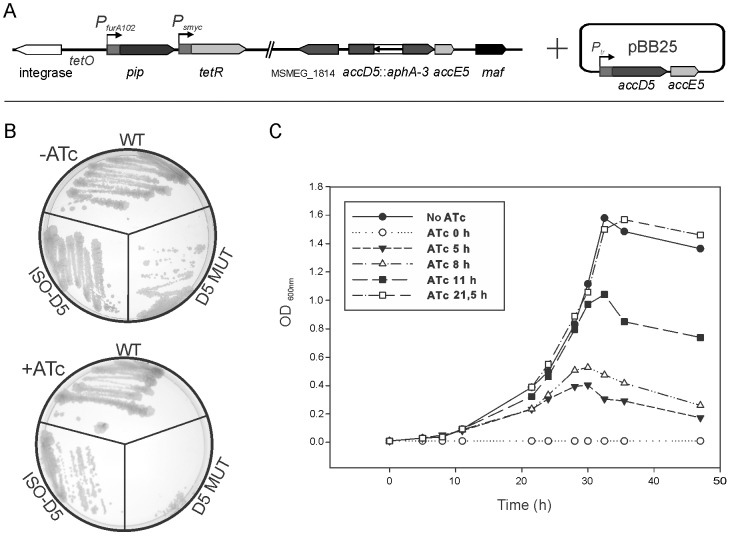
*accD5-accE5* conditional mutant D5 MUT. A) Schematic representation of the genetic organization of the *M. smegmatis* conditional mutant D5 MUT. In pBB25, transcription of the *accD5-accE5* operon is controlled by P*_tr_*, which can be repressed with the addition of ATc to the media. B) *M. smegmatis* mc^2^155 (WT), Isogenic (ISO-D5) and D5 MUT strains were grown on plates with 200 ng ml^−1^ (+ATc) or without ATc (−ATc). C) Growth curves of D5 MUT in the presence or absence of ATc 200 ng ml^−1^. A saturated culture of D5 MUT grown at 37°C was diluted in fresh 7H9 medium to an OD_600 nm_ of 0.01 and further incubated at 37°C. After 5, 8, 11 and 21 h; an aliquot of the main culture was separated and supplemented with ATc 200 ng ml^−1^. Growth was followed by measuring OD_600 nm_.

The conditional mutant D5 MUT, Fig. 3A, was constructed as described in Materials and Methods. Briefly, it contains an *accD5*::*aphA-3*/*accE5* intra-chromosomal allelic replacement, the integrative plasmid pFra42B carrying the TetR/Pip OFF system [38] and the replicative plasmid pBB25, which contains the wild type *accD5-accE5* operon under the transcriptional control of the pristinamycin (Pip)-dependent promoter P*_tr_*. In this strain, *accD5*-*accE5* expression can be down-regulated by the addition of anhydrotetracycline (ATc) to the media. When plated on solid media containing 200 ng ml^−1^ of ATc, the mutant D5 MUT was unable to grow, while the wild-type and the isogenic ISO-D5 (Materials and Methods) control strains grew normally under the same conditions, confirming that the *accD5-accE5* operon is essential for the viability of *M. smegmatis* (Fig. 3B).

In order to analyze the biochemical and metabolic consequences of reduced levels of AccD5 and AccE5 proteins in the D5 MUT mutant strain, we first followed the growth dynamic of the mutant in 7H9 liquid medium after the addition of ATc at different times. In the absence of ATc, condition where the target genes are constitutively expressed, the complemented mutant followed a growth dynamic very similar to the isogenic strain ISO-D5 (data not shown). In contrast, in the presence of ATc added at time zero, condition where the expression of the *accD5-accE5* operon is shut off, no cell growth could be detected (Fig. 3C), in agreement with the results obtained in solid medium (see above). Furthermore, the addition of ATc at early time points (5, 8 and 11 h) after inoculation led to a strong growth inhibition that was also reflected on the final ODs of the cultures (Fig. 3C). However, when ATc was added after the cells had entered exponential phase (e.g. at OD_600_>0.4, 21.5 h) there was no effect on the growth dynamic of the cultures. This result suggests that a lapse of time is needed to reduce the concentration of AccD5-AccE5 below a critical point to see an effect on growth; most likely as a result of several factors such as mRNA half-life or protein turnover. When ATc is added at higher ODs, cells must have synthesized enough amounts of protein, in the earlier stages of growth, to support the two or three rounds of replication needed to reach stationary phase.

**Figure 4:**
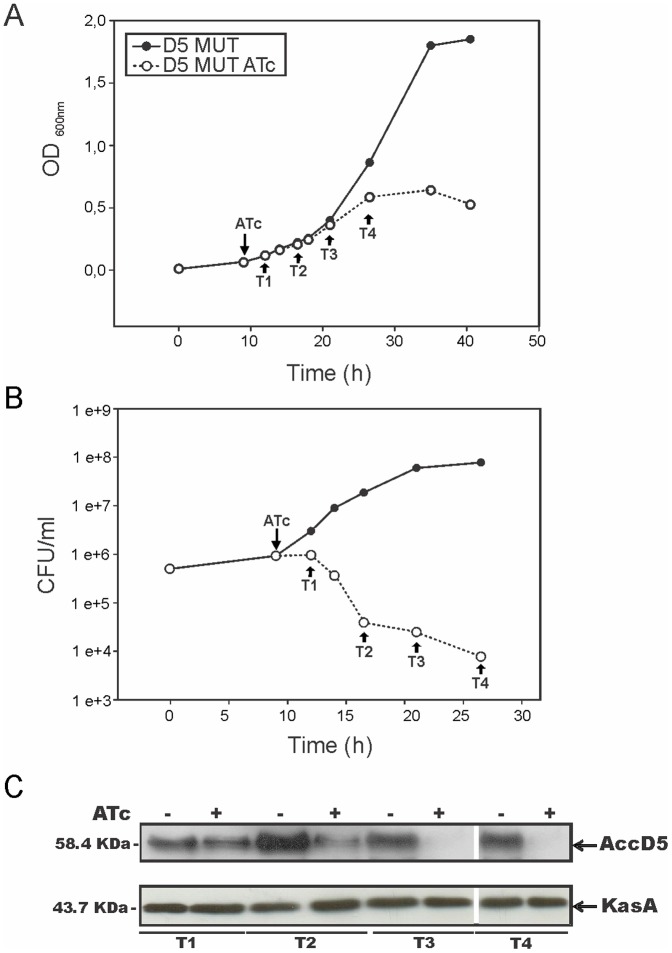
Effect of *accD5-E5* expression on D5 MUT growth and cell viability. A saturated culture of D5 MUT grown at 37°C was diluted in fresh 7H9 medium to an OD_600 nm_ of 0.01 and 9–10 h later (OD_600 nm_∼0,06) ATc 200 ng ml^−1^ was added to an aliquot of the culture. A) Growth was followed by measuring OD_600 nm_. Arrows indicate the times when aliquots of the cultures were collected for further analysis (T1, T2, T3 and T4). B) The number of viable cells of D5 MUT in the cultures grown in presence or absence of ATc was evaluated by plating serial dilutions onto LB plates at 37°C. C) Western blot analysis of total crude lysates from D5 MUT strain grown with (+) and without (−) ATc 200 ng ml^−1^. Detection was performed using anti-AccD5 antibodies elicited in rabbit (upper panel) and anti-KasA as loading control (lower panel).

On the basis of these results, we carried out our studies on the D5 MUT conditional mutant strain by growing the bacteria for 9–10 h (OD_600 nm_∼0.06) in 7H9 medium, and then dividing the culture into two equal fractions and supplementing one of them with ATc. Under these conditions, the mutant strain exhibited a typical exponential growth curve when grown in the absence of ATc, while in the presence of the antibiotic, the growth rate of the cultures slowed down until they stopped dividing at an OD_600 nm_∼0.6 (Fig. 4A). Furthermore, soon after the cultures entered stationary phase, the OD dropped steadily to reach an OD_600 nm_∼0.3, indicating a progressive lysis of the cells.

In order to correlate cell growth and expression of AccD5, protein levels were measured by Western blot after exposure to ATc. As shown in Fig. 4C, the reduced levels of *accD5* expression in the *accD5-accE5* conditional mutant treated with ATc led to a progressive decrease in AccD5 protein levels. As a control, Western blot using antibodies against the β-ketoacyl-acyl carrier protein synthetase KasA, involved in mycolic acid biosynthesis, showed that the levels of this protein did not change upon ATc treatment, confirming that the addition of ATc to the medium primarily affects the expression of the *accD5-accE5* operon. Remarkably, although the cells were still able to divide after ATc treatment, a large proportion of those cells could no longer duplicate and form a visible colony after being plated in solid medium (Fig. 4B). These results underline the essential nature of the ACCase 5 enzyme in the metabolism of *M. smegmatis* and establish a reliable platform to study the *in vivo* role of this enzyme complex.

### Effect of AccD5-AccE5 depletion in ACCase activities and on the acyl-CoA pool

**Figure 5:**
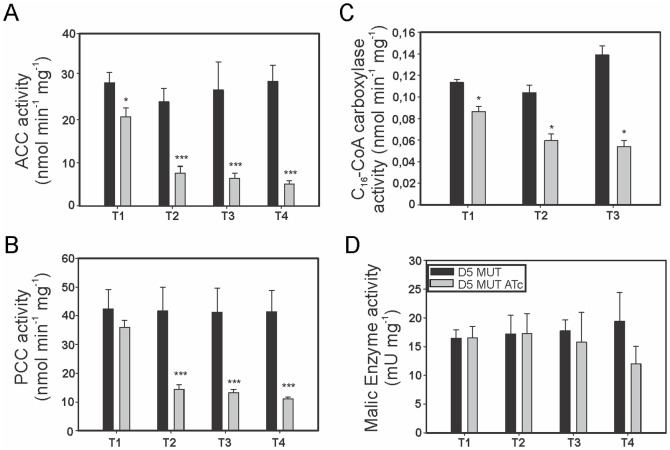
Acyl-CoA carboxylase activity in D5 MUT. Cell-free extracts were prepared from D5 MUT incubated in absence and presence of ATc 200 ng ml^−1^ at the times indicated in Fig. 4A. Determination of A) Acetyl-CoA carboxylase (ACC), B) Propionyl-CoA carboxylase (PCC), C) Long-chain acyl-CoA carboxylase and D) Malic enzyme activities. Levels of activity are the means of the results of three independent experiments ± standard deviations (n = 3). ***, *P* = 0.0001; *, P = 0.04.

In order to study the impact of the different levels of AccD5-AccE5 in the activity of the various acyl-CoA carboxylases present in *M. smegmatis*, cell-free extracts of the conditional mutant D5 MUT were prepared at different time points of the growth curve showed in Fig. 4A and assayed for PCC, ACC and long-chain ACCase activities (Figure 5A–C). The levels of ACC and PCC activities in D5 MUT were affected soon after ATc treatment showing up to 75% inhibition after ∼15 h (T3) in contrast with the steady levels of activity of the control culture (Fig. 5A–B). Strikingly, the level of long-chain (C_16_) acyl-CoA carboxylase activity was also reduced, but not as strongly as the ACC and PCC activities (Fig. 5C), suggesting that AccD5 and/or AccE5 also have a direct or indirect effect on the activity of the long-chain ACCase complex. To confirm that the loss in ACCase activities in the mutant strain was not related to a pleiotropic effect caused by a general reduction in the metabolic activity of the cultures in the presence of ATc, we assayed the cytoplasmic levels of the NADP-dependent malic enzyme. As shown in Fig. 5D, the levels of this enzyme at the earliest time points (T1–T3) were very similar compared with those obtained in cell-free extracts of non-treated cultures. This supports the notion that mutant cells depleted in AccD5-AccE5 remain metabolically active at least for ∼15 h after ATc treatment (T3). It is noteworthy that the malic enzyme activity was also significantly affected after ∼20 h of treatment (Fig. 5D, T4) suggesting that a late pleiotropic effect on the general metabolism of *M. smegmatis* is induced by the reduced levels of the ACCase activities.

**Figure 6:**
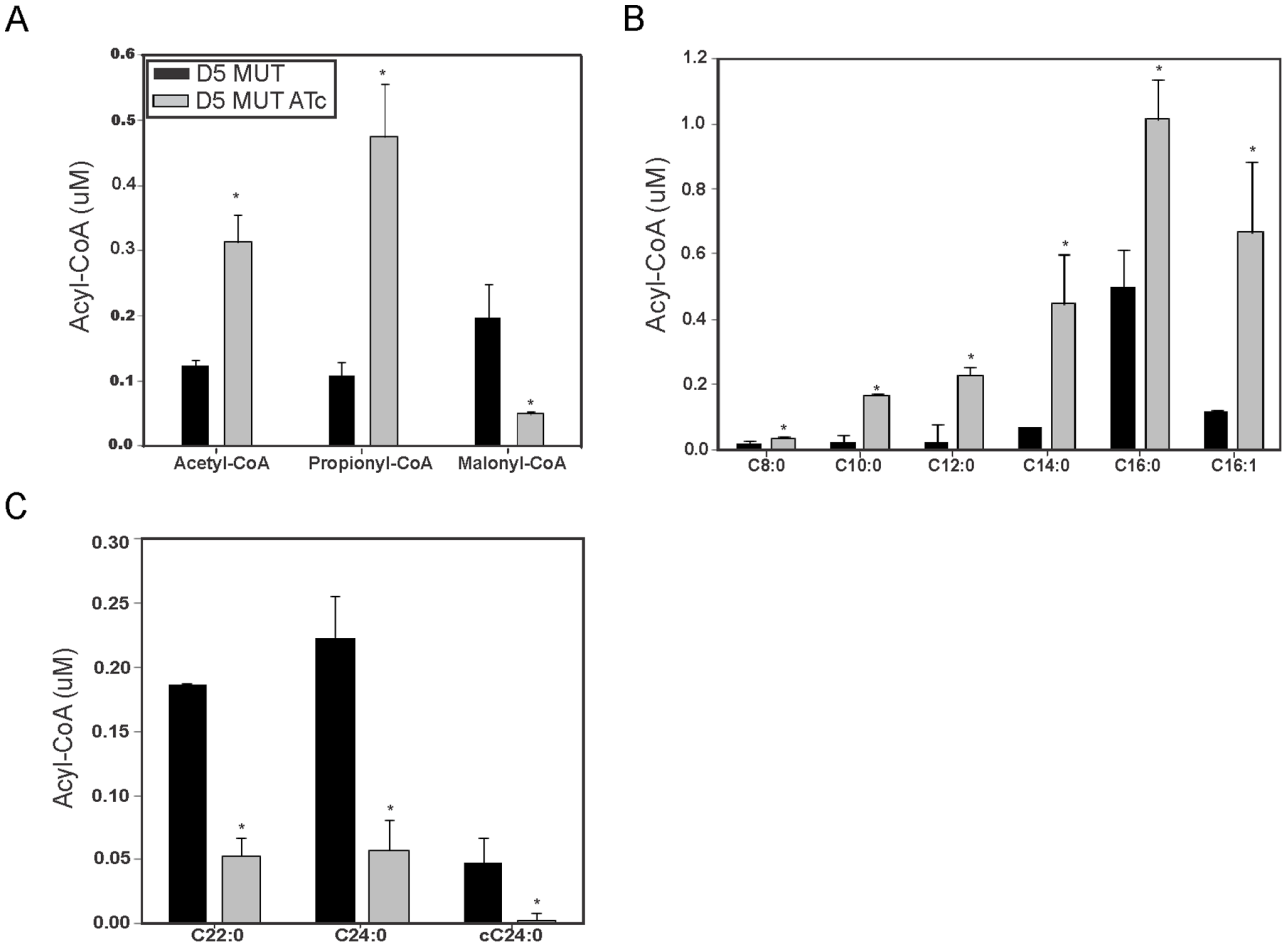
Acyl-CoA pool in D5 MUT. Acyl-CoAs were extracted from the same number of cells of cultures incubated in the absence or presence of ATc during ∼10 h (T2). Samples were analyzed by LC-MS. A) Short chain acyl-CoAs. B) Medium and long chain acyl-CoAs. C) Very long chain acyl-CoAs. Results are the means of three independent experiments ± standard deviations (n = 3). *, *P* = 0.04. cC24: carboxy-C_24_-CoA.

To further analyze the consequences of ACCase 5 depletion, we determined the acyl-CoA composition in the mutant strain in the presence or absence of ATc. Cultures were grown for 9 h, divided into two aliquots and one of them treated with ATc for ∼10 h (T2). Acyl-CoAs extracted from a fixed number of cells were analyzed by LC-MS. As shown in Fig. 6A, a clear increase in the content of acetyl-CoA and propionyl-CoA, with a concomitant decrease in the malonyl-CoA content, was observed in the cultures depleted of *accD5-accE5*. This result suggested that the main physiological function of ACCase 5 complex is that of ACC/PCC. Unfortunately, we were unable to correlate the increased levels of propionyl-CoA in the ATc treated cultures with reduced levels of methylmalonyl-CoA due to the comigration of this metabolite with its isomer succinyl-CoA (data not shown). Also, we observed that the lower level of malonyl-CoA impacted on the chain length of the complete repertoire of acyl-CoAs: there is a clear accumulation of medium to long chain acyl-CoAs (C_8_–C_16_) (Fig. 6A) and, in contrast, a reduction of the levels of very long-chain acyl-CoAs (C_22_–C_24_) (Fig. 6C). However, when we analyzed the levels of α–carboxyl-C_24_-CoA, key intermediate needed for the generation of mycolic acid by Pks13, we also observed a clear reduction of this metabolite in the ATc treated cultures (Fig. 6C). This effect could be a consequence of the reduced levels of C_24_-CoA but we cannot discard the hypothesis that AccD5 and AccE5 have an additional role in the carboxylation of C_24_-CoA together with the ACCase 4 complex.

### The absence of AccD5-AccE5 affects fatty acid and mycolic acid biosynthesis

**Figure 7:**
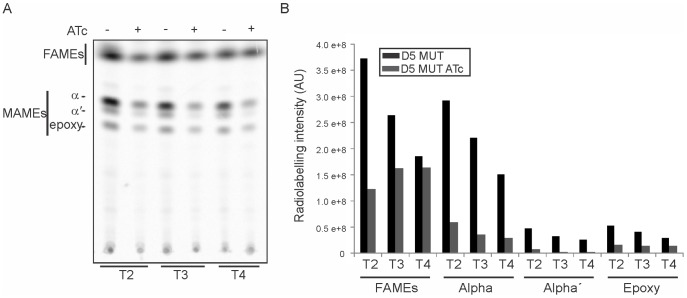
Fatty and mycolic acid profiles in D5 MUT. A) *de novo* fatty acid and mycolic acid biosyntheses. At the indicated times, aliquots from D5 MUT cultures incubated in absence or presence of ATc were labelled with [^14^C]-acetate for 1 hour at 37°C. Fatty acid and mycolic acids methyl esters were extracted from samples containing equivalent amounts of bacteria and were analyzed by TLC. B) Quantification of the radiolabelling intensity of the TLC showed in panel A.

To further understand the physiological role of the ACCase 5 complex, the lipid content of the D5 MUT conditional mutant treated or not with ATc was analyzed. Briefly, cultures were grown for 9 h, divided into two aliquots and one of them treated with ATc. At ∼10, 15 and 20 h after addition of ATc (T2, T3 and T4) both cultures were labeled for a period of one hour in the presence of [^14^C]acetate that incorporates into the neosynthesized fatty and mycolic acids. The fatty and mycolic acids methyl esters extracted from the same number of radiolabelled bacteria were analyzed by radio-TLC. As shown in Figure 7A, a significant decrease in the *de novo* fatty acid and mycolic acid biosyntheses was observed when the D5 MUT strain was exposed to ATc, and the inhibition level increased with time. These results are in agreement with the reduced ACC activity measured at these time points (Fig. 5A) and the strong decrease in the common elongation unit of FAS-I and FAS-II, malonyl-CoA (Fig. 6A). The biosynthesis of the three types of mycolic acids of *M. smegmatis*, α, α′ and epoxy, is similarly affected (Fig. 7A–B). Interestingly, the decrease in mycolic acid biosynthesis is considerably more pronounced than that on fatty acid biosynthesis (Fig. 7B and S3).

## Discussion

Considering the importance in providing the building blocks for fatty acid, mycolic acid and polyketide biosyntheses, the protein subunits of the ACCases involved in these processes are expected to be essential for the viability of mycobacteria. Studies carried out by high-density mutagenesis in *M. tuberculosis* indeed suggested that the genes encoding for one of the α subunits (*accA3*), three β subunits (*accD4*, *accD5*, *accD6*) and the å subunit (*accE5*) are essential for the viability of this microorganism [34], [35]. However, concluding genetic experiments directed to demonstrate the essentiality of these genes were only reported for *M. smegmatis accD6*, the gene encoding the CT subunit of ACCase 6 [21] and for the CT subunit of the ACCase 4 complex, AccD4 [23]. In this work, by using a classic two-step homologous recombination approach [36] and *in trans* complementation studies, we were able to determine unambiguously the essentiality of both *accD5* and *accE5* genes for *M. smegmatis* viability (Fig. 2).

To address the physiological role of AccD5 and AccE5 polypeptides, we constructed a conditional mutant of the *accD5-accE5* operon and named it D5 MUT. Phenotypic analysis of this mutant showed that the levels of ACC and PCC activities exhibited a dramatic decrease under reduced *accD5-accE5* expression conditions (Fig. 5A–B), and that this effect was reflected in a clear increase in the content of acetyl-CoA and propionyl-CoA, the substrates of the ACCase 5 complex, and a concomitant decrease in malonyl-CoA (Fig. 6A). The limited availability of this metabolite, used as elongation unit by both FAS-I and FAS-II systems, had a strong impact on the *de novo* biosyntheses of fatty acids and mycolic acids (Fig. 7A–B), as well as in the composition of the medium- to very long-chain acyl-CoA pools (Fig. 6B–C). The accumulation of C8 to C16 acyl-CoA could occur as a consequence of an inhibition of the elongase activity of FAS I. Interestingly, the previous characterization of an *accD6* conditional mutant also showed reduced levels of ACC and PCC activities and an inhibition of the *de novo* mycolic and fatty acid biosyntheses [21]. These results, together with the kinetics studies carried out *in vitro* for both enzyme complexes [17], [20], would suggest that ACCase 5 and ACCase 6 have overlapping biochemical and physiological roles. However, the absence of the AccD5 subunit cannot be complemented by AccD6, even under overexpression conditions of the latter (Fig. 2), implying that ACCase 5 and 6 have distinct roles *in vivo*.

It is worth noting the high sensitivity of *M. smegmatis* cells to having reduced levels of AccD5 (Fig. 4). The analysis of early time points after the addition of ATc (Fig. 4, T1 and T2) allowed us to determine that at those points the levels of AccD5 become lower than in the control strain (without ATc addition) and that the remaining amount of AccD5 let the cells duplicate at least one to two times. However, when those cells are plated on a solid media without the antibiotic they no longer form a visible colony; suggesting that they are either tagged for cell death (e. g. because they accumulate a toxic metabolite) or that they cannot revert the inhibition of the *accD5* expression by the ATc added to the cultures.

The subunit composition of the enzyme complex involved in the biosynthesis of the C_24_–C_26_ α-carboxy acyl-CoA in mycobacteria is still a matter of debate. By using anti-myc epitope antibodies, Portevin *et al* [23] were able to co-immunoprecipitate myc-tagged AccD4 with AccA3 and AccD5, suggesting that at least these three subunits might form a hetero-oligomeric enzyme complex *in vivo*. This result, together with the genetic studies that suggested that AccD4 is the CT component responsible of the generation of the C_24_–C_26_ α-carboxy acyl-CoA in mycobacteria would suggest that the ACCase complex responsible for the carboxylation of a long chain acyl-CoA physically interacts with AccD5 [23], [39]. However, these studies have not been validated through enzyme kinetic studies using purified proteins. The only biochemical data available was obtained by reconstituting a putative ACCase 4 complex with the AccA3 and AccD4 subunits; however the specific activity reported for this enzyme complex, using palmitoyl-CoA as a substrate, was at least 500 times lower than the one found for ACCases 5 and 6 [20]. Furthermore, our attempts to assay long-chain ACCase activity using different combinations of the AccA3, AccD4, AccD5 and AccE5 subunits and multiple assays conditions systematically failed (unpublished results). Further analysis of the D5 MUT mutant in the present work revealed a significant drop of the long-chain ACCase activity that followed a similar profile of the ACC/PCC activities after ATc treatment (Fig. 5C). As a consequence, the level of α–carboxy-C_24_-CoA was lower compared with the mutant strain grown without the addition of ATc (Fig. 6C), suggesting a putative role of the AccD5 and/or AccE5 subunits in the long-chain ACCase enzyme, which would be consistent with the immunoprecipitation data quoted above and would explain the inhibition of mycolic acid biosynthesis observed. Previous studies based on genetic and biochemical interaction experiments proposed a model in which at least three specialized FAS II complexes involved in mycolic acid biosynthesis are interconnected [40], [41]. The model predicts that a core complex formed by InhA, MabA and MtFabD is associated to specific condensing enzymes and dehydratases, defining their substrate specificity. We consider that the different ACCases present in mycobacteria could also be part of these complexes, having defined interactions with the specific complex to which they provide the substrate. Whether the additional role observed for AccD5-AccE5 in the long chain acyl-CoA carboxylase is functional or merely structural due to precise protein-protein interactions remains to be studied.

Our results pave the way towards understanding the biological roles of ACCases, in addition to providing a new target for rational development of antituberculosis therapeutics.

## Supporting Information

Figure S1 ***accD5*** **and** ***accE5*** **genes are part of a single transcriptional unit.** PCR analysis using primers D5-ms-final and FD5-Rv-Hind. Different templates of *M. smegmatis* mc^2^155 were assayed: 1, mid-log phase cDNA; 2, late-log phase cDNA: 3, genomic DNA (positive control); 4, mid-log phase RNA. 5, late-log phase RNA. 6, no template. MW: ladder 100 bp Molecular Weight Marker.
(PDF)

Figure S2 **Southern blot analysis of the** ***accD5-accE5*** **mutant D5 MUT.** Chromosomal DNA was digested with *Eco*RI and probed for hybridization with a labeled 390 bp fragment corresponding to the 5′ region of *accD5*. *M. smegmatis* mc^2^155 DNA (wt) and D5SCO6 (SCO) DNA were included as a control. Molecular masses are indicated in kilobases.
(PDF)

Figure S3 **Fatty and mycolic acid profiles in D5 MUT.** At the indicated times, aliquots from D5 MUT cultures incubated in absence or presence of ATc were labelled with [^14^C]-acetate for 1 hour at 37°C. Fatty acid and mycolic acid methyl esters were extracted from D5 MUT and equal amount of radioactivity (40000 cpm) were loaded in each lane.
(PDF)

File S1 **This file includes Table S1 (Plasmids used in this work), Table S2 (Bacterial strains used in this work) and the description of all plasmids construction.**
(DOCX)
