# Correction: Pleiotropic Effect of AccD5 and AccE5 Depletion in Acyl-Coenzyme A Carboxylase Activity and in Lipid Biosynthesis in Mycobacteria

**DOI:** 10.1371/journal.pone.0242528

**Published:** 2020-11-11

**Authors:** Bernardo Bazet Lyonnet, Lautaro Diacovich, Matías Cabruja, Fabienne Bardou, Annaïk Quémard, Gabriela Gago, Hugo Gramajo

After this article [1] was published, concerns were raised about results reported in Fig 4C: in both the AccD5 and KasA western blot panels, lanes 5 and 6 are duplicated in lanes 7 and 8.

**Fig 4:**
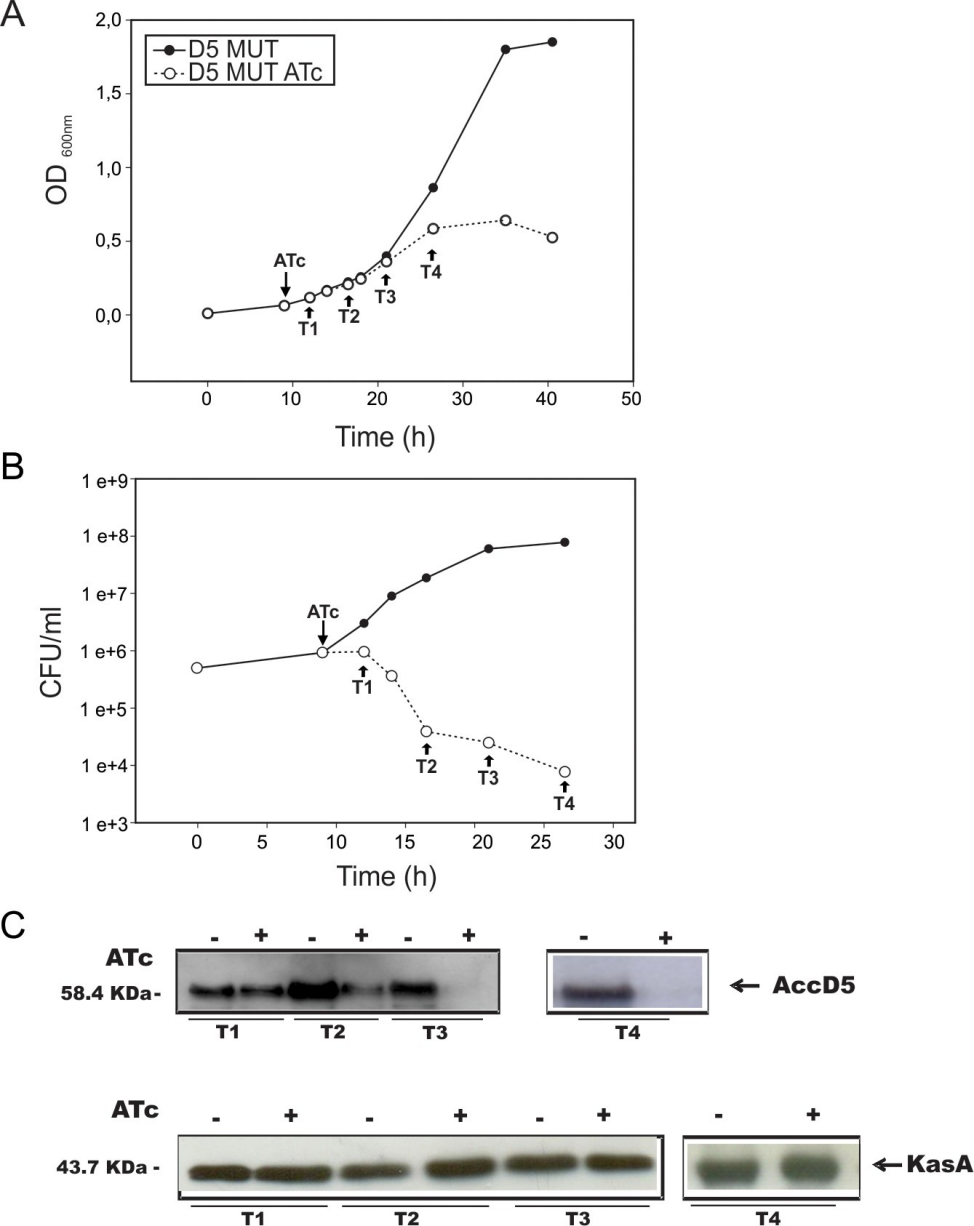
Effect of *accD5-E5* expression on D5 MUT growth and cell viability. A saturated culture of D5 MUT grown at 37°C was diluted in fresh 7H9 medium to an OD_600_ nm of 0.01 and 9–10 h later (OD_600_ nm∼0,06) ATc 200 ng ml^−1^ was added to an aliquot of the culture. A) Growth was followed by measuring OD_600_ nm. Arrows indicate the times when aliquots of the cultures were collected for further analysis (T1, T2, T3 and T4). B) The number of viable cells of D5 MUT in the cultures grown in presence or absence of ATc was evaluated by plating serial dilutions onto LB plates at 37°C. C) Western blot analysis of total crude lysates from D5 MUT strain grown with (+) and without (−) ATc 200 ng ml^−1^. Detection was performed using anti-AccD5 antibodies elicited in rabbit (upper panel) and anti-KasA as loading control (lower panel). The T4 data shown in panel C were obtained in a different western blot experiment than the T1-T3 data.

The authors apologize and note that these duplications arose due to errors in figure preparation. The results in Fig 4C included data obtained on two separate blots. In preparing the figure, lanes 7 and 8 of each panel were generated using data from the wrong blots.

A corrected Fig 4 is provided here; the original raw blot images for this experiment are in S1 File. Note that the T4 blot results were obtained in a different experiment than T1-T3 results, as is explained in the updated figure legend. The raw data and updated figure support the results reported in the figure: similar results were obtained for KasA in ATc-treated and untreated cells at all timepoints; Accd5 was expressed at lower levels in ATc-treated cells, and Accd5 expression decreased over time in ATc-treated samples.

The underlying data for other results reported in this article are provided in S2–S11 Files. The authors confirmed that the data supporting Fig 6 are available upon request.

## Supporting information

S1 File Raw western blot images underlying Fig 4C. Western blots of AccD5 and Kas A. Detection was performed using anti-AccD5 or anti-KasA antibodies elicited in rabbit. A) Raw data of AccD5 Western Blot. Lane 1, WT control, lanes 2 is empty and lanes 3 to 8 corresponding to T1 to T3 samples, with and without ATc (shown in Fig 4C of the paper). B) Raw data of AccD5 Western Blot. Lanes 1 to 6 correspond to samples from T2 to T4, with and without ATc. The last two lanes, corresponding to the T4 data, is now shown in a separate box in the new version of Fig 4C. C) Raw data of KasA Western Blot. Lanes 1 to 6 correspond to samples from T1 to T3, with and without ATc. (shown in Fig 4C); lane 7 corresponds to the wt strain used as control. D) Raw data of KasA Western Blot. Lanes 1 to 6 correspond to samples from T2 to T4, with and without ATc. T4 data is shown in a separate box in the new version of Fig 4C. In all the gel pictures (A, B, C, D) lane 1 is at the left.
(TIF)

S2 File Raw image underlying Fig 1D. (TIF)

S3 File Raw data underlying Fig 3C. (JNB)

S4 File Raw data underlying Fig 4A. (JNB)

S5 File Raw data underlying Fig 4B. (JNB)

S6 File Raw data underlying Fig 5A. (JNB)

S7 File Raw data underlying Fig 5B. (JNB)

S8 File Raw data underlying Fig 5C. (JNB)

S9 File Raw data underlying Fig 5D. (JNB)

S10 File Raw data underlying Fig 7A. (TIF)

S11 File Raw data underlying Fig 7B. (XLS)
