# Supplementary material for: Pleiotropic Effect of AccD5 and AccE5 Depletion in Acyl-Coenzyme A Carboxylase Activity and in Lipid Biosynthesis in Mycobacteria
Source: PLoS One. 2014 Jun 20;9(6):e99853. doi: 10.1371/journal.pone.0099853 (PMC4064979; doi:10.1371/journal.pone.0099853)
Supplement: Figure S1 — accD5 and accE5 genes are part of a single transcriptional unit. PCR analysis using primers D5-ms-final and FD5-Rv-Hind. Different templates of M. smegmatis mc2155 were assayed: 1, mid-log phase cDNA; 2, late-log phase cDNA: 3, genomic DNA (positive control); 4, mid-log phase RNA. 5, late-log phase RNA. 6, no template. MW: ladder 100 bp Molecular Weight Marker. (PDF) [file pone.0099853.s001.pdf]

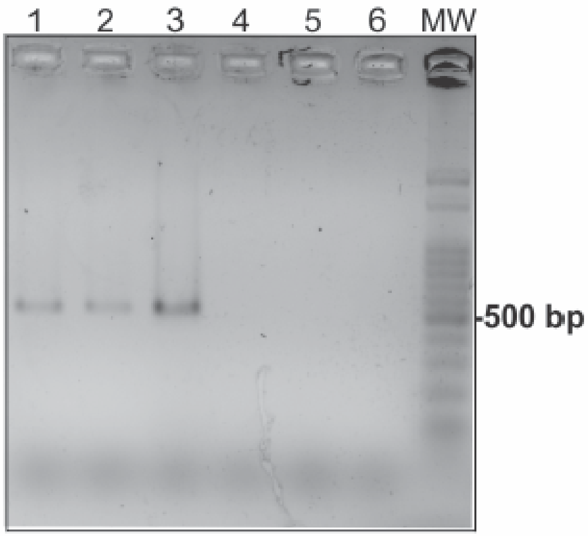

Figure S1. *accD5* and *accE5* genes are part of a single transcriptional unit. PCR analysis using primers D5-ms-final and FD5-Rv-Hind. Different templates of *M. smegmatis* mc^{2}155 were assayed: 1, mid-log phase cDNA; 2, late-log phase cDNA: 3, genomic DNA (positive control); 4, mid-log phase RNA. 5, late-log phase RNA. 6, no template. MW: ladder 100 bp Molecular Weight Marker.
