# Supplementary material for: Pleiotropic Effect of AccD5 and AccE5 Depletion in Acyl-Coenzyme A Carboxylase Activity and in Lipid Biosynthesis in Mycobacteria
Source: PLoS One. 2014 Jun 20;9(6):e99853. doi: 10.1371/journal.pone.0099853 (PMC4064979; doi:10.1371/journal.pone.0099853)
Supplement: Figure S2 — Southern blot analysis of the accD5-accE5 mutant D5 MUT. Chromosomal DNA was digested with EcoRI and probed for hybridization with a labeled 390 bp fragment corresponding to the 5′ region of accD5. M. smegmatis mc2155 DNA (wt) and D5SCO6 (SCO) DNA were included as a control. Molecular masses are indicated in kilobases. (PDF) [file pone.0099853.s002.pdf]

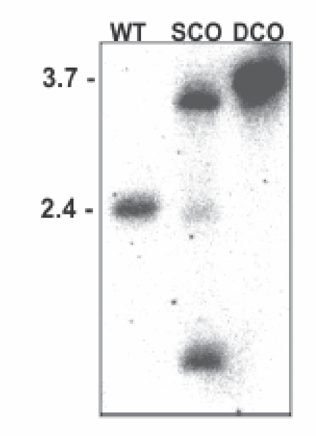

Figure S2. Southern blot analysis of the *accD5-accE5* mutant D5 MUT. Chromosomal DNA was digested with *Eco*RI and probed for hybridization with a labeled 390 bp fragment corresponding to the 5´ region of *accD5*. *M. smegmatis* mc^{2}155 DNA (wt) and D5SCO6 (SCO) DNA were included as a control. Molecular masses are indicated in kilobases.
