# Supplementary material for: Pleiotropic Effect of AccD5 and AccE5 Depletion in Acyl-Coenzyme A Carboxylase Activity and in Lipid Biosynthesis in Mycobacteria
Source: PLoS One. 2014 Jun 20;9(6):e99853. doi: 10.1371/journal.pone.0099853 (PMC4064979; doi:10.1371/journal.pone.0099853)
Supplement: Figure S3 — Fatty and mycolic acid profiles in D5 MUT. At the indicated times, aliquots from D5 MUT cultures incubated in absence or presence of ATc were labelled with [14C]-acetate for 1 hour at 37°C. Fatty acid and mycolic acid methyl esters were extracted from D5 MUT and equal amount of radioactivity (40000 cpm) were loaded in each lane. (PDF) [file pone.0099853.s003.pdf]

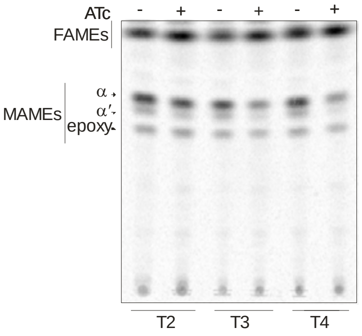

Figure S3. Fatty and mycolic acid profiles in D5 MUT. At the indicated times, aliquots from D5 MUT cultures incubated in absence or presence of ATc were labelled with [^{14}C]-acetate for 1 hour at 37°C. Fatty acid and mycolic acid methyl esters were extracted from D5 MUT and equal amount of radioactivity (40000 cpm) were loaded in each lane.
