# Supplementary material for: Pleiotropic Effect of AccD5 and AccE5 Depletion in Acyl-Coenzyme A Carboxylase Activity and in Lipid Biosynthesis in Mycobacteria
Source: PLoS One. 2014 Jun 20;9(6):e99853. doi: 10.1371/journal.pone.0099853 (PMC4064979; doi:10.1371/journal.pone.0099853)
Supplement: File S1 — This file includes Table S1 (Plasmids used in this work), Table S2 (Bacterial strains used in this work) and the description of all plasmids construction. (DOCX) [file pone.0099853.s004.docx]

Table S1. Plasmids used in this work.

| Plasmids | Relevant genotype and/or information | Reference/source |
| --- | --- | --- |
| pMP349 | *E. coli-Mycobacterium* shuttle vector, Apra^r^ | [[1](#_ENREF_1)] |
| pBB25 | pMP349 harboring *M. smegmatis accD5-accE5* genes under P*_tr_* promoter control, Ap^r^ | This study |
| pPR27 | *E. coli-Mycobacterium* shuttle vector, *oriM* temp^s^, *sacB, xylE*, Gm^r^ | [[2](#_ENREF_2)] |
| pPR-FD5 | pPR27 derivative carrying *accD5-accE5*∷*aphA-3*, Gm^r^ Km^r^ | This study |
| pCG76 | *E. coli-Mycobacterium* shuttle vector, *oriM* temp^s^, Str^r^ | [[3](#_ENREF_3)] |
| pCG-FD5 | pCG76 derivative harboring *M. smegmatis accD5* plus 500 pb upstream and downstream., Str^r^ | This study |
| pFra42B | pFRA40 derivative; P*_smyc_*-*tetR*(→); P*_furA102_tetO-pip*(→)*;* P*_ptr_-lacZ*; *int*; Str^r^ | [[4](#_ENREF_4)] |
| pMP395 | *E. coli-Mycobacterium* shuttle vector, Apra^r^ | [[1](#_ENREF_1)] |
| pBB42 | pMP395 harboring *M. smegmatis accD5* under P_GroEL_ promoter control, Apra^r^ | This study |
| pBB45 | pMP395 harboring *M. smegmatis accE5* including 51 pb upstream of the GTG initiation codon, under P_GroEL_ promoter control, Apra^r^ | This study |
| pBB46 | pMP395 harboring *M. smegmatis accE5* under P_GroEL_ promoter control, Apra^r^ | This study |
| pBB47 | pMP395 harboring *M. smegmatis accD5-accE5* under P_GroEL_ promoter control, Apra^r^ | This study |
| pCGHD6 | pCG76 derivative harboring *accD6* plus 500 bp, both upstream and downstream of this ORF from *M. smegmatis*, Str^r^ | [[5](#_ENREF_5)] |

Gm^r^*,* gentamicin resistance*;* Km^r^, kanamycin resistance; Apra^r^ , apramycin resistance; Str^r^, streptomycin/ spectinomycin resistance

Table S2. Bacterial strains used in this work.

| Strain | Relevant genotype and/or information | Reference/source |
| --- | --- | --- |
| *E. coli* |  |  |
| DH5α | *E. coli K12 F- ΔlacU169 (φ80lacZΔM15) endA1 recA1 hsdR17 deoR supE44 thi-1λ- gyrA96 relA1* | [[6](#_ENREF_6)] |
| *M. smegmatis* |  |  |
| mc^2^155 | Electroporation-proficient *ept* mutant of *M. smegmatis* strain mc26 | [[7](#_ENREF_7)] |
| D5SCO6 | *M. smegmatis* mc^2^155 harboring pPR-FD5 integrated into the *accD5* locus, Km^r^ | This study |
| D5DCO2 | *M. smegmatis accD5* conditional mutant strain harboring pCG-FD5, Km^r^ St^r^ | This study |
| D5 MUT1 | *M. smegmatis accD5* conditional mutant strain harboring pFra42B and pBB25, Km^r^ St^r^ Apra^r^ | This study |
| ISO-D5 | *M. smegmatis* harboring pFra42B and pBB25, Str^r^ Apra^r^ | This study |

Gm^r^*,* gentamicin resistance*;* Km^r^, kanamycin resistance; Apra^r^ , apramycin resistance; Str^r^, streptomycin/ spectinomycin resistance

Supporting Information

Plasmid construction

pBB25. The DNA fragment containing the P*_tr_* promoter was obtained from pFra50 [[4](#_ENREF_4)] digested with *Eco*RI and *Pvu*II and cloned into *Eco*RI*/Pvu*II-cleaved pMP349, yielding pBB22. The *accD5-accE5* genes from *M. smegmatis* mc^2^155 were PCR amplified from genomic DNA using the oligonucleotides D5-ms-Nsi-UP (5´-GTGACCCATGCATACGAGPCGTTAC-3´), to introduce an *Nsi*I site (underlined) at the translational start codon of *accD5*, and FD5-Rv-Hind (5´-AAGCTTGACGACGCCGAACC-3´). The PCR product was digested with *Nsi*I and *Xba*I and cloned into *Nsi*I*/Xba*I-cleaved pBB22, yielding pBB25.

pPR-FD5. The complete *accD5* gene from *M. smegmatis*, including 322 bp upstream and 442 bp downstream of this ORF, was PCR amplified from genomic DNA using the oligonucleotides FD5-Fw-Xba (5´-TCTAGACCAGTGGCAGCAAAAGA-3´) and FD5-Rv-Hind (see above). The PCR product was cloned into pCR-Blunt II-TOPO^®^, yielding pTOPO-FD5. This plasmid was digested with *Xba*I and *Hind*III and the *accD5* containing fragment was cloned into the *Xba*I*/Hind*III sites of pET28a(+), yielding pET-FD5. The *aphA-3* cassette that confers Km resistance was obtained from pUC4K as a 1.2 kb *Bam*HI fragment and cloned into a *Bam*HI-cleaved pET-FD5, yielding pET-FD5∷*km*. Finally, pPR-FD5, the construct used for allelic exchange, was obtained by transferring the *Xba*I*-Not*I fragment from pET-FD5∷*km* containing the *accD5∷aphA-3* mutated allele into *Xba*I*-Not*I*-*cleaved pPR27, a temperature sensitive mycobacterial vector carrying the counter-selectable marker *sacB* and the *xylE* reporter gene.

pCG-FD5. pTOPO-FD5 was digested with *Xba*I and *Spe*I and the fragment containing *accD5* plus 322 bp upstream and 442 bp downstream of this ORF was cloned into a *Xba*I-cleaved pCG76 [[3](#_ENREF_3)], yielding pCG-FD5.

pBB42. The P*_GroEL_* promoter was obtained from pVV2 [[8](#_ENREF_8)] as an *Xba*I*-Hind*III fragment and cloned into the *Xba*I*/Hind*III-cleaved pMP395 [[1](#_ENREF_1)], yielding pBB40. The *accD5* gene from *M. smegmatis* mc^2^155 was PCR amplified from genomic DNA using the oligonucleotides D5Ms-rbs*Nde*I (5´-AAGGAGATATCATATGACGAGCGTTTACCGAGCCG-3´), to introduce an *Nde*I site (underlined) at the translational start codon of *accD5*, and D5Ms-Hind (5´-GCAAGCTTTCACAGCGGAATGTTCCCGTG-3´). The PCR product was digested with *Nde*I and *Hind*III and cloned into *Nde*I*/Hind*III-cleaved pBB40, yielding pBB42.

pBB45. The *accE5* gene from *M. smegmatis* mc^2^155 containing 51 bp upstream of the translation start site of this gene was PCR amplified from genomic DNA using the oligonucleotides E5-ms-UP-Nde (5´-CGACATATGAGCGGCGCGAAC-3´) and FD5-Rv-Hind (5´-AAGCTTGACGACGCCGAACC-3´). The PCR product was digested with *Nde*I and *Hind*III and cloned into *Nde*I*/Hind*III-cleaved pBB40, yielding pBB45.

pBB46. The *accE5* gene from *M. smegmatis* was PCR amplified from genomic DNA using the oligonucleotides E5-ms-chica-UP-*Nde*I (5´-AGCGGCCATATGCAGGCCGACG-3´) and FD5-Rv-Hind (5´-AAGCTTGACGACGCCGAACC-3´). The PCR product was digested with *Nde*I and *Hind*III and cloned into *Nde*I*/Hind*III-cleaved pBB40, yielding pBB46.

pBB47. The *accD5*-*accE5* genes from *M. smegmatis* mc^2^155 were PCR amplified from genomic DNA using the oligonucleotides D5Ms-rbsNdeI (5´-AAGGAGATATCATATGACGAGCGTTTACCGAGCCG-3´), to introduce an *Nde*I site at the translational start codon of *accD5* and FD5-Rv-Hind (5´-AAGCTTGACGACGCCGAACC-3´). The PCR product was digested with *Nde*I and *Hind*III and cloned into *Nde*I*/Hind*III-cleaved pBB40, yielding pBB47.

References

1. Consaul SA, Pavelka MS, Jr. (2004) Use of a novel allele of the *Escherichia coli aacC4* aminoglycoside resistance gene as a genetic marker in mycobacteria. FEMS Microbiol Lett 234: 297-301.

2. Pelicic V, Jackson M, Reyrat JM, Jacobs WR, Jr., Gicquel B, *et al.* (1997) Efficient allelic exchange and transposon mutagenesis in *Mycobacterium tuberculosis*. Proc Natl Acad Sci USA 94: 10955-10960.

3. Guilhot C, Otal I, Van RI, Martin C, Gicquel B (1994) Efficient transposition in mycobacteria: construction of *Mycobacterium smegmatis* insertional mutant libraries. J Bacteriol 176: 535-539.

4. Boldrin F, Casonato S, Dainese E, Sala C, Dhar N, *et al.* (2010) Development of a repressible mycobacterial promoter system based on two transcriptional repressors. Nucleic Acids Res 38: e134.

5. Kurth DG, Gago GM, Bazet LB, Lin TW, *et al.* (2009) Accase 6 is the essential acetyl-CoA carboxylase involved in fatty acid and mycolic acid biosynthesis in mycobacteria. Microbiology.

6. Hanahan D (1983) Studies on transformation of *Escherichia coli* with plasmids. J Mol Biol 166: 557-580.

7. Snapper SB, Melton RE, Mustafa S, Kieser T, Jacobs WR, Jr. (1990) Isolation and characterization of efficient plasmid transformation mutants of *Mycobacterium smegmatis*. Mol Microbiol 4: 1911-1919.

8. Dhiman RK, Schulbach MC, Mahapatra S, Baulard AR, Vissa V, *et al.* (2004) Identification of a novel class of omega,E,E-farnesyl diphosphate synthase from *Mycobacterium tuberculosis.* J Lipid Res 45: 1140-1147.
