# Supplementary figures and images for: Correction: Pleiotropic Effect of AccD5 and AccE5 Depletion in Acyl-Coenzyme A Carboxylase Activity and in Lipid Biosynthesis in Mycobacteria
Source: PLoS One. 2020 Nov 11;15(11):e0242528. doi: 10.1371/journal.pone.0242528 (PMC7657537; doi:10.1371/journal.pone.0242528)

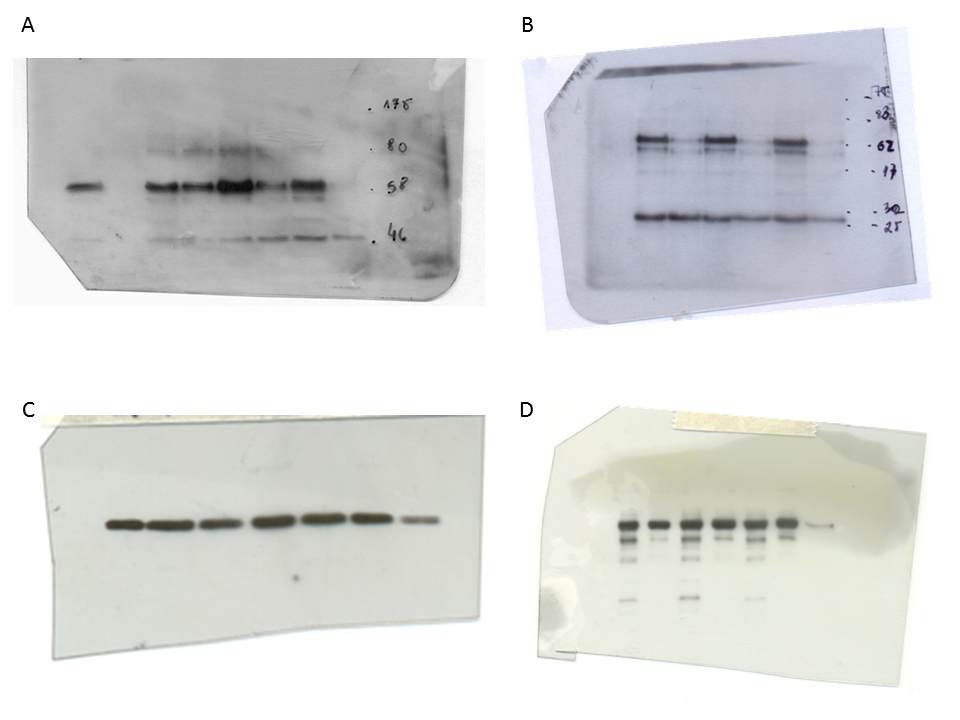

Supplement: S1 File — Western blots of AccD5 and Kas A. Detection was performed using anti-AccD5 or anti-KasA antibodies elicited in rabbit. A) Raw data of AccD5 Western Blot. Lane 1, WT control, lanes 2 is empty and lanes 3 to 8 corresponding to T1 to T3 samples, with and without ATc (shown in Fig 4C of the paper). B) Raw data of AccD5 Western Blot. Lanes 1 to 6 correspond to samples from T2 to T4, with and without ATc. The last two lanes, corresponding to the T4 data, is now shown in a separate box in the new version of Fig 4C. C) Raw data of KasA Western Blot. Lanes 1 to 6 correspond to samples from T1 to T3, with and without ATc. (shown in Fig 4C); lane 7 corresponds to the wt strain used as control. D) Raw data of KasA Western Blot. Lanes 1 to 6 correspond to samples from T2 to T4, with and without ATc. T4 data is shown in a separate box in the new version of Fig 4C. In all the gel pictures (A, B, C, D) lane 1 is at the left. (TIF) [file pone.0242528.s001.tif]
